# Quality Parameters of Spanish Lemons with Commercial Interest

**DOI:** 10.3390/foods10010062

**Published:** 2020-12-29

**Authors:** Marlene G. Aguilar-Hernández, Dámaris Núñez-Gómez, María Ángeles Forner-Giner, Francisca Hernández, Joaquín J. Pastor-Pérez, Pilar Legua

**Affiliations:** 1 Department of Horticulture, Universidad Nacional Agraria La Molina, Av. La Molina s/n, 15026 Lima, Peru; maguilarhe@lamolina.edu.pe; 2 Department of Plant Sciences and Microbiology, Escuela Politécnica Superior de Orihuela (EPSO), Miguel Hernandez University, Ctra. de Beniel, Km. 3,2, 03312 Orihuela, Spain; francisca.hernandez@umh.es (F.H.); p.legua@umh.es (P.L.); 3 Citriculture and Vegetal Production Center, Valencian Institute for Agricultural Research, Apartado Oficial, 46113 Moncada, Spain; forner_margin@gva.es; 4 Departmento de Ingeniería Agroforestal, Escuela Politécnica Superior de Orihuela (EPSO), Miguel Hernandez University, Ctra. de Beniel, Km. 3,2, 03312 Orihuela, Spain; jjpastor@umh.es

**Keywords:** sugars, antioxidants, phenols, *Citrus limon* (L.) Burm f., rootstock

## Abstract

The Spanish Mediterranean region concentrates the largest producers of lemons (*Citrus limon* Burm. f.) at the national level where the 98.4% of the cultivated area of lemons corresponds to the varieties “Verna” and “Fino”. In this study, the morphological and chemical variations of the fruits obtained in five variety/rootstock combinations were investigated in order to determine the influence and impact of the rootstock on the physicochemical properties of the fruits. The assay was carried out using three lemon varieties (“Fino 95”, “Fino 49” and “Verna”) grafted onto two different rootstocks (*Citrus macrophylla* and *Citrus aurantium*). The varieties were selected due to be consolidated commercial varieties, while the rootstocks are the most commonly used in the world. Both the morphological characteristics of the fruits (colour, weight, size) as well as their physicochemical characteristics (total soluble solids, titratable acidity, maturity index, antioxidant activity, sugars, and organic acids) were evaluated. Based on the results, the lemons with the best physicochemical and the best compositional characteristics were obtained in the “Fino 95” and “Fino 49” lemons grafted onto *C. aurantium* rootstock presented the highest quality fruits.

## 1. Introduction

The lemon (*Citrus limon* Burm f.) represents one of the most important citrus fruits in the world after the orange and the mandarin, for its various food, industrial and medicinal/therapeutic uses [1,2]. Worldwide, Spain holds the second position in the ranking among the lemon-producing countries, with its production concentrated in the southeast of the country, that is, in the Mediterranean region [3]. In this region, among the most relevant varieties of Spanish origin of lemon trees, the varieties “Verna” and “Fino” stand out. These two varieties concentrate 98.4% of the cultivated area of lemons in Spain.

The “Fino” lemons (autumn-winter variety) are of high quality for international exports to European countries from October to February when prices are higher, while the “Verna” season runs from March to July when prices on the market are low [4].

The importance of adequate rootstocks in the cultivation of citrus fruits is vital for the development of the plantation. Citrus rootstocks affect many external and internal characteristics of the fruit, such as size, shape, skin thickness, juice content, total soluble solids, and phytonutrient composition. In recent times, many efforts have been made to search and evaluate new rootstocks that are better adapted to different areas and environmental conditions [4,5,6,7,8,9,10]. However, in citrus cultivation, it is necessary to carry out new research aiming to identify the impact, behaviour and synergies between the variety and the rootstock mainly related to its resistance/adaptability (water stress, nutrients, soil biota among others), harvest yield per tree, and morphological and quality fruit characteristics. The selection of one rootstock to the detriment of another one can give to the citric farmers a competitive advantage, or not, since the correct choice can facilitate and improve crop management, the trees can show better resistance/adaptability to different agro-environmental variations, and the fruit obtained can present the characteristics physicochemical demanded by both the market and consumers.

Historically, the bitter orange tree (*Citrus aurantium* L.) has been the most used rootstock in lemon trees. However, this rootstock is highly sensitive to the Citrus Tristeza Virus (CTV) [11] and is being replaced by the *Citrus macrophylla* rootstock, due to its greater vigour, which induces higher yields and an earlier harvest than with *C. aurantium* [6].

When selecting a rootstock, adaptability to the prevailing soil conditions and the horticultural characteristics of the cultivar, such as tree growth, yield and, above all, fruit quality, are considered. The objective of this study was to compare the quality of the fruit obtained from the principal bloom, from the morphological and physicochemical point of view as well as the antioxidant capacity of three commercial varieties of lemon (“Fino 95”, “Fino 49” and “Verna”) grafted on two rootstocks: *C. macrophylla* and *C. aurantium*.

Once the importance of the study of the variety/rootstock interactions for lemon cultivation has been highlighted, it is striking that, to our knowledge, there are no specific studies with these variety/rootstock combinations, since they represent the main lemon cultivation combinations in Spain.

## 2. Materials and Methods

### 2.1. Plant Material and Sampling

In the present study, three commercial varieties of *Citrus limon* (“Verna”, “Fino 95” and “Fino 49”) grafted on two different rootstocks (*C. macrophylla* and *C. aurantium*) were used (Table 1). The 10-year-old trees were on located in a commercial lemon plantation in Alicante, Spain. The growing (drip irrigation with addition of NPK 4-1-1.5 fertilizer) and soil conditions (clay soil, 44.4% CaCO_3_, 17.1% active calcium carbonate, 5.79 mS cm^−1^ and pH = 8.5) were maintained the same for all varieties and rootstocks. All the conditions (environmental, soil, irrigation, fertilization, climate, etc.) remained homogeneous for all the trees studied in order to evaluate the morphological and nutritional variations/differences. among the three varieties grafted onto two rootstocks. The fruits were collected in the middle of May for “Verna” combinations and the beginning of October for “Fino” combinations according to the commercial collected dates. In all the cases, the lemon fruits resulted from the principal bloom and all the trees were in perfect sanitary conditions. Representative samples of the fruits were manually collected with specific pruning shears. Twenty fruits from five trees for each combination (*n* = 5) were collected with a total of 100 lemons. The fruits collection was made in all tree orientations, internal and external, aiming to avoid the edge effect. For this study, “Fino 95” grafted on *C. aurantium* was not considered because it is not a commercial combination and it is not used in commercial plantations. This is due to “Fino 95” being a very early variety and the *C. aurantium* rootstock delays its maturation.

### 2.2. Morphological Characterization of the Fruits

The assays were carried out on the same day of harvesting on fruit held at room temperature. The size of the fruits was determined by the measuring the equatorial diameter (ED), polar diameter (PD), size of the neck and base nipple by means of a digital caliper (model 500-197-20, 150 mm; Mitutoyo Corp., Aurora, IL, USA). To determine the peel thickness (PT), the lemon fruits were split vertically in the area of the equator, later the measurements were taken at equidistant points by a digital caliper (model 500-197 -20, 150 mm; Mitutoyo Corp., Aurora, IL, USA). The weight of the fruits was also determined (model AG204 scale; Mettler Toledo, Barcelona, Spain) with a precision of 0.1 mg. Additionally, the number of seeds and segments of each of the fruits was counted for all the samples, by means of simple visual manual counting.

### 2.3. Colour Determination

Colour was measured using a colorimeter (model CR-300, Minolta, Osaka, Japan). Four readings of the peel were made in four equatorial and equidistant zones of all the fruits. Measurements were made at constant room temperature (22 ± 2 °C). Colour was evaluated according to the Commission Internationale de l’Éclairage (CIE) and expressed as L*, a* and b* colour values [12]. The coordinates L*, a* and b* indicate the lightness of the colour (L* = 0 and L* = 100 represent the colour white and black, respectively), its position between green and red (the negative and positive values of a* indicate green and red, respectively) and its position between blue and yellow (negative and positive values of b* indicate blue and yellow, respectively) [13]. The target colour (C* = (a* 2 + b* 2) 1/2), the hue angle (H⁰ = arctan (b*/a*), and colour index (CI = a* × 1000/L* × b*) were also calculated.

### 2.4. Biochemical Characterization of the Fruits

Biochemical analyses were determined on five juice samples (3 lemons each) for each variety/rootstock combination studied. The juice was carefully obtained using a commercial manual juicer and immediately centrifuged at 15,000 rpm for 20 min (Sigma 3-18 K, Osterode and Harz, Lerbach, Germany). The juice samples were kept in the freezer at −18 °C until later analysis for pH, titratable acidity (TA) and total soluble solids (TSS) were calculated according to the methodology described by Aguilar-Hernández et al., (2020). The maturity index (MI) was calculated as the TSS/TA ratio. All analyses were carried out at constant room temperature (22 ± 2 °C).

### 2.5. Organic Acids and Sugars Content

Sugar and organic acids in lemon juices were determined according to methodology described by Legua et al. [14] with using the Agilent 1100 high performance liquid chromatography (HPLC) with ChemStation software. Sugar and organic acids were separated using a Supelcogel C610H column, 30 cm × 7.8 mm, and a Supelguard pre-column, 5 cm × 4.6 mm (Supelco, Bellefonte, PA, USA). A diode array detector (DAD) (Diode Aray DAD G1315A, Bellefonte, PA, USA) set at 210 nm and a refractive index detector (RID) (G-1362-A) were used to quantify organic acids and sugars, respectively. In both cases, reference standards were used for the organic acids (L-ascorbic acid, malic acid, citric acid, oxalic acid, acetic acid, lactic acid and succinic acid) and the sugars (glucose, fructose and sucrose) supplied by Sigma Aldrich (Poole, Dorsert, UK) with calibration curves with R^2^ ≥ 0.999. The results obtained are expressed in g 100 mL^−1^.

### 2.6. Total Polyphenols and Antioxidant Activity

The antioxidant activity (AA) was studied using three different but widely used methodologies recognized in the bibliography, such as the 2.2′-Azinobis [3-ethylbenzothiazolin-6-sulfonic] radical method (ABTS^+^), the 2.2′-Diphenyl-1-Picrylhydrazyl radical method (DPPH^•^) and by reduction of the ferric ion (FRAP) by means of a UV-visible spectrophotometer (Hellos Gama 9423 Uvg 1002E model, Thermo Spectonic, Gloucester, UK) according to the methodology described by Wojdyło et al. [15]. The results were expressed in mmol Trolox per liter of juice as the mean value of the triplicate repetitions. The total polyphenols content (TPC) was determined by means of the Folin-Ciocalteau colorimetric reagent as described by Singleton et al. [16]. Readings were made by spectrophotometry (HP 8451, Cambridge, UK) compared to the gallic acid calibration curve. Tests were carried out in triplicate and the results are presented as mg gallic acid equivalents (GAE) per 100 mL of juice.

### 2.7. Statistical Analysis

The significant differences of the data were evaluated by means of the one-way analysis of variance (ANOVA) followed by the Tukey’s multiple range test for p < 0.05. Statistical analysis was performed with the Minitab Statistical Software 19 data analysis software.

## 3. Results

### 3.1. Fruit Quality Parameters

In most of the parameters studied, significant differences were observed between varieties and rootstocks (Table 2). The highest fruit weight (183.6 g) was obtained for the V-M lemons, while the lemons of “Verna” variety grafted on *C. aurantium* (V-A) showed the lowest values (100.1 g). In relation to the size of the fruits, the maximum and minimum values were 66.9 mm (V-M) and 55.0 mm (V-A) for equatorial diameter (ED) and 91.9 mm (F95-M) and 72.2 (V-A) for polar diameter (PD), respectively. In this way, considering the fruit weight (FW) and its size (ED), the varieties grafted on *C. macrophylla* showed the highest values compared to the varieties grafted on *C. aurantium* (V-M > F49-M > F95-M > F49-A > V-A). All the fruits presented a similar geometry regardless of the cultivar and/or rootstock with ED/PD values between 0.7 and 0.8, where the most elongated shape was observed only in F95-M (0.6). Despite the fact that all the lemons studied had a juice percentage > 31%, F95-M and F49-A had the highest percentages, 44.3% and 41.6%, respectively, while V-A had the lowest values (31.7%). The highest and lowest number of segments in the fruits were observed for F49-M (10.2) and V-A (8.8) respectively, while the rest of varieties presented values > 9 (F49-A > F95-M > V-M). Significant differences were observed in relation to the number of seeds contained in the lemons, with maximum values of 11.3 for F49-M and minimum values of 1.3 for F95-M.

### 3.2. Physical-Chemical Parameters of the Fruits

Both the colour of the peel and the juice of the different lemons were studied (Table 3). The luminosity (L*) in the fruit peel ranged from 68.1 (V-A) to 64.3 (F95-M), but the differences were not significant between the three varieties grafted on *C. macrophylla* and *C. aurantium*. The F95-M lemons (3.9) were the only ones that presented red coloration (positive values of a*), while for the other varieties/rootstock the coloration was green (negative values of a*) with the maximum and minimum for F49-A (−7.5) and VM (−2.6) respectively. The b* values were statistically homogeneous in all the fruits, going from more to less yellow (positive values of b*) F95-M > F49-A > F49-M > V-A > V-M. In the same line, the color index (CI) values did not show statistically significant differences between the samples, presenting values between −2.5 (F49-A) and 1.1 (F95-M), which corresponds to shades between yellow-green (more negative values) and pale yellow (values close to 0).

Likewise, the brightest juice (L*) ranged from 75.1 to 56.9 for three cultivars grafted on *C. macrophylla* and *C. aurantium* rootstocks, but were not significantly different. The a* values showed two different groups, on the one hand, F49-M (6.6) and F95-M (4.8) with positive values of a* indicating reddish coloration, and on the other, F49-A (−7.6), VA (−6.20) and VM (−5.2) with greenish coloration indicated by the negative values of a*.

All the juices presented yellowish coloration (positive b* values) with F49-M > F95-M > F49-A > V-A > V-M from more to less yellow. In relation to the CI, V-M (−3.1), F49-A (−3.0), and V-A (−2.7) showed yellowish-green tones while the tone for F49-M (1.5) and F95-M (1.0) was pale yellow.

The varieties “Verna” and “Fino 49”, both with the *C. aurantium* rootstocks, presented the highest levels of total soluble solids contained in the juice (10.5° and 10.3 °Brix, respectively) while the lowest amount was for VM (8.4 °Brix). On the other hand, the cultivar “Fino 49” presented the highest TA values in both grafts, being 74.9 g citric acid L^−1^ for F49-A and 74.7 g citric acid L^−1^ for F49-M. The MI for all varieties/rootstocks studied was between 1.05 and 1.5 (Table 4).

### 3.3. Organic Acids and Sugars

Table 5 shows the content of sugars and organic acids of lemons obtained from the different variety/rootstock combinations studied. In all the fruits, glucose and fructose were identified as the main sugars, with values between 3.1 and 2.2 g 100 mL^−1^ and 4.0 and 2.7 g 100 mL^−1^ for glucose and fructose respectively. 

Regarding the organic acids, F49- no significate differences were observed for citric acid, while for malic acid, the differences were observed only between the varieties “Fino” and “Verna”. On the other hand, F95-M presented the maximal values for ascorbic acid (0.08 g 100 mL^−1^) statistically significative when compared with the other combinations of variety/rootstock. 

### 3.4. Total Polyphenol Content and Antioxidant Activity

The total polyphenols content was statistically homogeneous for all samples, that is, without significant differences between them, being that the maximum values were reported for VA (331.6 mg GAE 100 mL^−1^) and the minimum for F49-M (226.4 mg GAE 100 mL^−1^) (Table 6). 

Regarding the total antioxidant activity, of the three different methodologies used for its determination, the FRAP method did not show statistically significant differences between the samples, with values between 1.4 mmol Trolox L^−1^ (F95-M) and 1.1 mmol Trolox L^−1^ (VM). For the ABTS^+^ methodology, the data were statistically homogeneous in the range of 5.7–6.5 mmol Trolox L^−1^ for all samples except for F95-M (2.8 mmol Trolox L^−1^) which presented significant differences among the others. Finally, the antioxidant activity obtained by the DPPH^+^ method showed statistically different values only between “Verna” variety grafted on the two studied rootstocks being 3.0 and 1.2 mmol Trolox L^−1^ for V-M and V-A, respectively.

## 4. Discussion

The importance of appropriate rootstocks in the citrus fruits industry is well defined [5,17,18,19,20] but the results must be analysed with caution, since they undoubtedly establish and define the effects of rootstocks in different locations and for different commercial varieties of lemons, and may present significant variations between varieties, regions and growing conditions. However, the identification of the physicochemical and quality parameters of the lemons carried out in this study is adequate to evaluate and identify the potential differences/impacts of the variety/rootstock combinations studied, once the trees were grown under homogeneous conditions (climatic, cultivation, edaphic, nutritional, etc.) and the fruits came from the principal bloom. Note that the variations between the fruits of different blooms can be significant, despite being the same tree and the same agro-environmental conditions.

### 4.1. Fruit Quality Parameters 

The size of the fruit is an important parameter for citrus producers, where, based on this parameter, producers decide whether the fruit will be supplied to the market for consumption as fresh products or will be destined to the manufacturing and/or processing industry. Small-sized fruits are mainly processed for juice, although they can be consumed fresh. Medium to large fruit is the one that generally presents the highest profitability in the market for fresh consumption [5]

In this study, all the lemons of the variety/rootstock combinations obtained, except V-A, can be considered medium/large fruits, the largest being those of the V-M combination. Similar results were reported in the bibliography, where the fruits of the “Verna” variety had larger sizes when grafted to *C. macrophylla* than to *C. aurantium* [6]. In relation to the quality parameters of the fruit, although the highest amounts of juice were obtained in F95-M, the little difference with F49-A, together with the lower thickness of the skin, as well as the more rounded shape of the fruit, point out F49-M as the most appropriate, in relation to its fresh consumption and/or as juice in the industry. It should be noted that the results, both in relation to the amount of juice and the thickness of the skin, obtained in this study are higher and lower, respectively, when compared to the bibliography [4,6,21]. All the combinations studied obtained a quantity of juice higher than the minimum content (20%) required by the legislation for their commercialization [22].

The F95-M and V-A varieties proved to be the most appropriate combinations to produce fruits with fewer seeds and segments, a characteristic that makes them more palatable for fresh consumption. Another relevant parameter in relation to the quality of the fruits is the peel thickness, not being commercially attractive the extremes values. Thus, thick-rind fruits generally show little juice yield, while thin-rind fruits are more vulnerable to breakage and disruption during transport and/or storage [19]. In this study, the results indicated the combinations F95-M and F49-A with the finest rind, 5.8 mm and 4.9 mm respectively, coinciding with the fruits with the highest juice yield (44.3% in F95-M and 41.6% in F49-A). In all cases, the values obtained for the thickness of the rind were higher than those obtained in “Fino” and “Verna” lemons grown in other rootstocks, such as Forner-Alcaide 5, Forner-Alcaide 13 and Forner-Alcaide 517 [10], indicating the influence of the rootstocks on the quality of the fruit obtained.

### 4.2. Physicochemical Parameters of the Fruits

The flavour and palatability of citrus is a function of the relative levels of TSS, acids, and the presence or absence of various aromatic or bitter components of the juice [23]. The concentration of soluble solids in lemon juice should not be ignored as an important parameter, although the fruit quality standards for lemons do not include a minimum requirement for this [22]. The amount of TSS of the fruits of the three varieties studied in *C. macrophylla* presented the highest values (F49-M > V-M > F95-M). However, in this study, the results obtained for TSS in the juices of all variety/rootstock combinations were higher than those defined in the specific bibliography, established between 6–7.5 °Brix for the variety “Verna” [24] and between 6.5–9.2 °Brix for the variety “Fino” [21] possibly due to significant differences in edaphic and/or climatological conditions or even the influence of the rootstocks.

On the other hand, titratable acidity (TA) is used as an indicator of citrus juice quality and is also useful to determine the appropriate harvest time for production practices [25]. In this study, the TA values of the lemon juices “Fino 95”, “Fino 49” and “Verna” did not show significant differences between the grafts used, and for all variety/rootstocks combinations the values are shown within the expected range [26].

The external colouring of the fruit (colour of the peel) is generally associated with the internal quality (flavour and texture), which can become a decisive factor for the consumer and, therefore, its price in the market. The change in colour from green to yellow in lemon fruits is associated with alterations in the composition and concentration of pigments, mainly chlorophylls and carotenoids. When the air temperature falls below 13 °C, the degradation of chlorophylls begins at the same time as the synthesis of carotenoids starts, which are the compounds responsible for the yellow colour [27]. Based on the results (Table 3), the little or no influence of the rootstock on the final colour of the juice could be affirmed, since the values of a* and H ° were similar and did not present significant differences. These results are in agreement with those obtained by other authors who already indicated the limited influence of the rootstock on the colour of the fruits [4,28,29]. This parameter would be much more influenced by other factors, such as temperature, humidity, and solar incidence among others [27,29,30,31].

### 4.3. Organic Acids and Sugars

Total acidity is considered a relevant factor in the general quality of the juice, as well as in determining the moment of harvest [19]. In this study, four organic acids with direct influence on the acidity of lemons were identified: citric acid, malic acid, ascorbic acid, and succinic acid.

Despite being the main acid identified with percentages between 73% and 80% of the total organic acids, citric acid did not show significant differences between the variety/rootstock combinations studied. The results were superior when compared with those indicated for lemons of the varieties “Verna” and “Fino 49” obtained in different rootstocks Forner-Alcaide [10], although they were consistent when compared to other varieties of *Citrus limon* [32,33].

Malic acid, commonly present in some fruits such as apples, bananas, pears, and plums, has been identified in citrus fruits as a secondary acid substitute for citric acid [34]. In general, the content of malic acid identified for all the variety/rootstocks combinations studied was considerably higher than those identified for both other lemon varieties such as Eureka, which ranged between 0.17 and 0.26 g 100 mL^−1^ [35], and for the same varieties obtained in a different rootstock [10], which provides the variety/rootstocks combinations studied a soft and acid flavour without impact on the taste in the mouth [36].

Ascorbic acid (vitamin C) presented concentrations between 490 and 840 mg L^−1^ in F95-M and VM respectively, showing values considerably higher than those for “Fino” and “Verna” lemons [24,37] as well as other varieties [38,39]. However, despite the quantitative difference, the results obtained are shown in accordance with the bibliography, where higher values of L-ascorbic acid had already been reported in “Fino” lemons than in “Verna” lemons [24,26]. These variations may be related to the clone of the variety, the maturity of the fruits and the climatic and edaphic conditions of the crop. Nevertheless, can be also related to the differences derivate the different blooms of the trees, even be the same tree and under the same cultivation conditions. Ascorbic acid is used as an antioxidant to prevent the damage of free radicals and other reactive oxygen species [40], it influences many metabolic processes such as gene expression and cell division, defence reactions and intestinal absorption of iron among others [40,41,42], in addition to being an important antioxidant traditionally used in the food, pharmaceutical and/or cosmetic industry [43,44]. Therefore, identifying and quantifying varietal differences can be of great commercial and industrial interest.

Along the same lines as ascorbic acid, succinic acid or butanedioic acid, presents high commercial interest, since it can be used in the pharmaceutical industry (i.e., biostimulant and anticoagulant), food industry (i.e., antioxidant E363) and agriculture (i.e., growth regulators for plants and insecticide) among others [45,46]. In this sense, the identification of natural sources with high concentrations of succinic acid is important both from an industrial and economic point of view. In this study, significant differences were identified between the variety/rootstocks combinations studied, however, the succinic acid values obtained were considerably higher than those reported in other varieties [10,47] indicating its potential use in the industry as well as the influence of both the variety and the rootstock used to obtain the fruits.

In relation to the glucose and fructose concentrations identified in this study, results were observed in line with other investigations. Likewise, Aguilar et al. [10] reported similar concentrations of fructose and glucose, although slightly lower, for the varieties “Fino 49” and “Verna” obtained in different rootstocks Forner-Alcaide, confirming the influence of the rootstock on the content of sugars in the fruits. Albertini et al. [33] highlighted fructose as the predominant sugar in “non-acid” varieties of citrus fruits, while for acid varieties it would be glucose, confirming the results obtained in this study for all the variety/rootstocks combinations analysed.

Since the metabolism of sucrose is dependent on the enzymes β-fructosidase, for the synthesis of fructose, and α-glusodidase, for glucose [48], it could be stated that the varieties studied present a higher activity of β-fructosidase versus α-glusodidase, mainly in the fruits of F49-M and VA, where the highest fructose/glucose ratio (0.8) was obtained. This enzymatic predominance related to the content of fructose and glucose in the lemon fruits can be confirmed since other studies did not identify the direct impact of the rootstock on the sugar content but rather related it mainly to the harvest flowering [49]. However, for all the lemons studied, the fructose/glucose ratio, which varied between 0.71 and 0.80, did not reach the range of variation indicated by the European Fruit Juice Association, established between 0.95 and 1.3 [22]. These results reconfirm the influence of both the variety and the rootstock on the predominant sugar metabolic pathway, as proposed by Oustric et al. [50].

### 4.4. Total Polyphenol Content and Antioxidant Activity

Different investigations demonstrate the great variety of beneficial biological effects of phenolic compounds that include hepatoprotective, anti-inflammatory, anticancer and antibacterial actions among others [51,52,53,54], in addition to contributing to the sensory and organoleptic quality of fruits due to its influence on parameters such as colour, astringency and/or flavour. In our case, the total content of polyphenols was similar for the five variety/rootstock combinations studied with a range between 226.4 ± 44.1 to 331.6 ± 44.9 mg GAE 100 mL^−1^. These values are lower than those reported for “Verna” and “Fino” obtained in Forner-Alcaide rootstocks [10] but higher than those reported for the Eureka variety [47]. Other studies indicate the influence of the method used for the juice extraction, as a possible reason for the variation in the total polyphenol content, since the peel of lemons has higher levels compared to the pulp and/or juice [55,56]. The higher content of phenolic compounds in the fruit peel may be related to the protective effect against degradation derived from the incidence of ultraviolet light, pathogens, and predators [57].

The antioxidant capacity determined by the FRAP, DPPH^•^ and ABTS^+^ methods showed considerably lower results than those reported for both “Fino” and “Verna” determined during three consecutive seasons in fruits grown in an area close to that of this study [24,58], only “Fino 49” presented ABTS^+^ values significantly higher than those reported by González-Molina et al. [58] possibly due to the effect of environmental parameters such as irrigation, fertilization, temperature, complementary treatments for the management and/or control of pests, etc. In general, the antioxidant capacity for all the studied Spanish variety/rootstock combinations was much higher than that obtained for different Italian and Chinese varieties [59,60], indicating the excellent antioxidant capacity of the variety/rootstock combinations analysed.

## 5. Conclusions

Morphological and biochemical characteristics of the fruits among the five variety/rootstock combinations studied were quite consistent and similar. The cultivar “Fino 49” stands out as opposed to “Verna”, and presented better physicochemical and compositional characteristics. In relation to the rootstock, the results indicated *C. aurantium* as the rootstock that presented the highest quality fruits (juice percentage, peel tightness, an appropriate size for fresh consumption, ascorbic and malic acid, fructose/glucose ratio). Based on the results obtained in this study, the relationship and/or influence existing between the variety/rootstocks combinations and the morpho chemical characteristics of the fruits can be confirmed in a limited way. It is difficult to clearly identify which is the degree of impact that corresponds to the root and which to the cultivar, in addition to considering the influence of environmental and/or cultivation conditions. However, thanks to this type of study, it is possible to determine and establish which variety/rootstocks combinations grown in a certain region may be more commercially interesting depending on their use (fresh consumption, juice, extract, etc.). Improvements in production, and therefore in the quality of the marketed fruits, can be developed considering the results obtained, providing scientific-technical advice on the maintenance and increase of the production of lemons.

## Figures and Tables

**Table 1:** Varieties and rootstocks studied in this work.

Cultivar	Rootstock	Acronym
“Fino 95”	*Citrus macrophylla*	F95-M
“Fino 49”	*Citrus macrophylla*	F49-M
“Fino 49”	*Citrus aurantium*	F49-A
“Verna”	*Citrus macrophylla*	V-M
“Verna”	*Citrus aurantium*	V-A

**Table 2:** Morphological characterization of lemons (“Fino 95”, “Fino 49” and “Verna”) obtained in different rootstocks (*C. macrophylla* and *C. aurantium*), with emphasis on the weight fruit (FW), equatorial diameter of the fruit (ED), polar diameter of fruit (PD), Peel thickness (PT), Number of fruit segments (NSG), Number of seeds (NSD) and percentage of juice (PJ). The values represented are the mean.

Parameter	F95-M	F49-M	F49-A	V-M	V-A
FW (g)	144.9 b	169.6 b	138.1 a	183.6 a	100.1 c
ED (mm)	61.0 b	66.5 a	59.9 b	66.9 a	55.0 c
PD (mm)	91.9 a	86.4 ab	77.5 c	84.9 b	72.7 c
ED/PD	0.6 b	0.7 a	0.7 a	0.8 a	0.8 a
PT (mm)	5.8 ab	6.4 a	4.9 b	6.7 a	6.8 a
NSG	9.8 ab	10.2 ab	9.8 ab	9.3 a	8.8 b
NSD	1.3 c	5.0 bc	11.3 a	7.8 ab	2.6 c
PJ (%)	44.3 a	36.7 bc	41.6 ab	36.8 bc	31.7 c

The different letters within the rows indicate significant differences according to the Tukey test (*p* < 0.05).

**Table 3:** Variations in the colour of the skin and the juice of lemons (“Fino 95”, “Fino 49” and “Verna”) obtained in different rootstocks (*C. macrophylla* and *C. aurantium*), where L* represents the luminosity, a* the green/red, b* blue/yellow, C* the chroma values, H° the hue angle and CI (color index) the citrus colour index. The values represented are the mean with their standard deviation in parentheses.

Parameter	F95-M	F49-M	F49-A	V-M	V-A
Colour of the peel					
L*	64.3 (7.3) a	64.6 (5.5) a	65.9 (3.7) a	65.6 (4.5) a	68.1 (4.8) a
a*	3.9 (4.6) a	−4.8 (3.1) b	−7.5 (2.0) b	−2.6 (3.7) a	−3.3 (2.7) b
b*	48.5 (8.8) a	44.7 (4.7) a	47.2 (3.2) a	42.4 (4.8) a	44.4 (3.7) a
C*	49.1 (7.5) a	45.1 (4.5) a	47.8 (3.0) a	42.7 (4.6) a	44.6 (3.6) a
H°	88.0 (21.1) a	96.5 (4.4) a	99.2 (2.7) a	93.9 (5.4) a	94.4 (3.6) a
CI	1.1 (1.4) a	−1.8 (1.4) a	−2.5 (0.8) a	−1.1 (1.5) a	−1.2 (1.0) a
Colour of the juice					
L*	74.7 (1.2) a	75.1 (1.9) a	61.6 (6.5) b	56.9 (10.4) a	61.3 (9.0) a
a*	4.8 (1.3) a	6.6 (2.4) a	−7.6 (2.6) a	−5.2 (2.7) a	−6.2 (3.0) a
b*	59.4 (1.9) a	59.9 (1.4) a	43.8 (7.0) a	38.1 (12.6) a	44.0 (10.9) a
C*	59.6 (2.0) c	60.3 (1.5) c	44.6 (6.8) c	38.6 (12.4) c	44.6 (10.7) c
H°	85.4 (1.1) a	83.7 (2.3) a	100.2 (3.5) a	99.0 (5.0) a	98.8 (4.5) a
CI	1.0 (0.2) a	1.5 (0.6) a	−3.0 (1.1) b	−3.1 (1.9) b	−2.7 (1.6) b

The different letters within the rows indicate significant differences according to the Tukey test (*p* < 0.05).

**Table 4:** Variations of total soluble solids (TSS), titratable acidity (TA) and maturity index (MI) of lemons (“Fino 95”, “Fino 49” and “Verna”) obtained in different rootstocks (*C. macrophylla* and *C. aurantium*). The values represented are the mean with their standard deviation in parentheses.

Parameter	F95-M	F49-M	F49-A	V-M	V-A
TSS (°Brix)	9.1 (0) b	7.8 (0.1) c	10.3 (0.1) a	8.4 (0.4) b	10.5 (0.1) a
TA (g citric acid L^−1^)	67.6 (3.5) b	74.7 (6.8) b	74.9 (1.4) a	56.0 (0.5) c	68.3(3.9) b
MI	1.3 (0.07) a	1.05 (0.08) b	1.3 (0.04) a	1.5 (0.07) a	1.5 (0.08) a

The different letters within the rows indicate significant differences according to the Tukey test (*p* < 0.05).

**Table 5:** Variations of sugars (glucose and fructose) and organic acids (citric acid, malic acid, ascorbic acid and succinic acid) of lemons (“Fino 95”, “Fino 49” and “Verna”) obtained in different rootstocks (*C. macrophylla* and *C. aurantium*). The values represented are the mean with their standard deviation in parentheses.

Parameter	F95-M	F49-M	F49-A	V-M	V-A
Sugars					
Glucose (g 100 mL^−1^)	3.0 (0.1) ab	2.2 (0.3) b	2.8 (0.08) ab	2.9 (0.3) ab	3.1 (0.4) a
Fructose (g 100 mL^−1^)	4.0 (0.08) a	2.7 (0.5) b	3.9 (0.07) a	3.6 (0.4) ab	3.8 (0.5) a
Organic acids					
Citric acid (g 100 mL^−1^)	5.7 (0.02) a	5.6 (0.5) a	6.3 (0.09) a	5.8 (0.3) a	5.4 (0.4) a
Malic acid (g 100 mL^−1^)	0.8 (0.09) a	0.8 (0.1) a	0.9 (0.004) a	0.4 (0.09) b	0.5 (0.1) b
Ascorbic acid (g 100 mL^−1^)	0.08 (0.001) a	0.05 (0.01) b	0.05 (0.003) b	0.04 (0.01) b	0.05 (0.004) b
Succinic acid (g 100 mL^−1^)	1.03(0.06) ab	0.7 (0.1) b	0.9 (0.05) ab	0.8 (0.1) ab	1.4 (0.4) a

The different letters within the rows indicate significant differences according to the Tukey test (*p* < 0.05).

**Table 6:** Variations of the total content of polyphenols and the total antioxidant activity of lemons (“Fino 95”, “Fino 49” and “Verna”) obtained in different rootstocks (*C. macrophylla* and *C. aurantium*). The values represented are the mean with their standard deviation in parentheses.

Parameter	F95-M	F49-M	F49-A	V-M	V-A
TPC (mg GAE 100 mL^−1^)	240.0 (44.1) a	226.4 (16.8) a	307.2 (59.0) a	281.1 (23.9) a	331.6 (44.9) a
Total antioxidant activity					
ABTS^+^(mmol TroloxL^−1^)	2.8 (0.5) b	5.7 (0.3) a	6.5 (1.0) a	6.2 (1.0) a	6.4 (0.4) a
DPPH^+^(mmol Trolox L^−1^)	1.7 (0.8) ab	2.0 (0.3) ab	2.0 (0.56 ab	3.0 (0.5) a	1.2 (0.09) b
FRAP (mmol Trolox L^−1^)	1.4 (0.2) a	1.1 (0.09) a	1.4 (0.1) a	1.1 (0.08) a	1.3 (0.2) a

The different letters within the rows indicate significant differences according to the Tukey test (*p* < 0.05).

## Abbreviations

ED	Equatorial diameter
PD	polar diameter
PT	peel thickness
FW	weight fruit
NSG	Number of fruit segments
NSD	Number of seeds
PJ	percentage of juice
CIE	*Commission Internationale de l’Éclairage*
CI	colour index
TA	titratable acidity
TSS	total soluble solids
MI	maturity index
HPLC	liquid chromatography
RID	refractive index detector
AA	antioxidant activity
ABTS^+^	2.2′-Azinobis [3-ethylbenzothiazolin-6-sulfonic] radical method
DPPH^•^	2.2′-Diphenyl-1-Picrylhydrazyl radical method
TPC	total polyphenols content
AGE	gallic acid equivalents
ANOVA	analysis of variance
